# Experiences and needs of older adults at different stages of cerebral infarction based on trajectory theory—A qualitative study

**DOI:** 10.1002/nop2.1398

**Published:** 2022-11-02

**Authors:** Xianping Tang, Hong Sun, Song Ge, Shuyu Han, Ying Li, Bei Wu

**Affiliations:** ^1^ School of Nursing Xuzhou Medical University Xuzhou China; ^2^ Department of Nursing Huaian Maternity and Child Healthcare Hospital Affiliated to Yangzhou University Medical College Huaian China; ^3^ Department of Natural Sciences University of Houston‐Downtown Houston Texas USA; ^4^ School of Nursing Fudan University Shanghai China; ^5^ NYU Rory Meyers College of Nursing New York New York USA

**Keywords:** cerebral infarction, long‐term illness trajectory, older adults, qualitative study

## Abstract

**Background:**

In recent years, stroke has become the second leading cause of death worldwide, and the incidence and mortality of ischemic stroke have increased significantly. This study mainly aimed to explore the experiences and needs of older adults at different stages of cerebral infarction based on the chronic illness trajectory theory.

**Methods:**

Data were collected from 22 older adults experiencing the onset, acute, and stable stages of stroke through semi‐structured interviews and were analyzed using Colaizzi's descriptive phenomenological approach.

**Results:**

Multiple themes and subthemes emerged on the experiences and needs of older adults at different stages of cerebral infarction based on the three dimensions of the long‐term disease trajectory theory: illness‐related work, biographical work, and everyday life work. Seven themes were extracted for illness‐related work, six for biographical work, and eight for everyday life work.

**Discussions:**

The treatment, nursing, and rehabilitation of cerebral infarction are complex. This study indicated that patients after cerebral infarction have different experiences and needs for illness‐related work. They also have distinctive and dynamically changing demands for biographical work and everyday life work.

**Conclusions:**

The experiences and needs of older patients with cerebral infarction changed dynamically at different stages of the disease. Healthcare professionals should develop effective interventions targeting these needs at various disease stages, provide patients with continuous support to shape their disease trajectories, and maintain patients' stability.

## 1 INTRODUCTION

Cerebral infarction, also known as ischemic stroke, accounts for 85% of all strokes globally (Jia, 2018; Katan & Luft, 2018). Although the global mortality and morbidity associated with stroke have shown an overall downward trend in the past 20 years, and the age‐adjusted stroke‐associated mortality in China has been reduced by 42.6%, stroke is a leading cause of morbidity and mortality in the world and in China (GBD, 2019; Wang et al., 2019). In 2016, China accounted for 40% of the 13.7 million new strokes and one‐third of the 5.5 million deaths associated with stroke in the world. In addition, China accounted for one‐third of the 116.4 million global stroke‐related disability‐adjusted life years (GBD, 2019). Specifically, the mortality rate of cerebral infarction among Chinese older adults has increased rapidly (Wang, Peng et al., 2020). The prevalence of disability is still as high as 75%, with severely disabled people accounting for more than 40% of disability (Li, Xiong et al., 2018). Traditional medical care approaches focus on alleviating patients' signs and symptoms during hospitalization but not focus meeting on their psychological, physical and long‐term care needs during rehabilitation. Studies have shown that high‐quality long‐term care could effectively reduce the risk of recurrence and disability in older adults, decrease their readmission rate and healthcare costs and promote their physical recovery and quality of life (Davoody et al., 2016; Qing, 2017; Watson et al., 2015). However, researchers need to learn more about older patients' needs and experiences to design more tailored, appropriate and effective interventions.

The long‐term disease trajectory theory is a comprehensive care model that includes long‐term care, health education, quality monitoring, reference and appointments. It integrates home care and case management to help patients adapt to daily life and improve their quality of life. Corbin and Strauss (1991) proposed the long‐term disease trajectory theory, a theoretical framework for developing tailored nursing interventions for patients at different stages of long‐term diseases (Corbin, 1998). This theory aimed to help patients prevent recurrence, manage symptoms, reduce complications, maintain a stable mental state and improve their quality of life. This theory divides the disease process into the following stages: pre‐trajectory, trajectory onset, crisis, acute, stable, unstable, comeback, downward and dying. Each stage has three dimensions of the theory, including illness‐related work, biographical work and everyday life work, which can be used for evaluation and intervention (Corbin, 1998). Illness‐related work refers to the treatment and management of long‐term diseases and complications, such as managing signs, symptoms and complications and avoiding risks. Biographical work is an individual's self‐awareness in the process of life, affected by his/her stage of life and coping ability. There are four basic processes of biographical work including integrating the disease into life, adapting to the disease and its consequences, re‐establishing an individual's own concept and changing his/her future. Everyday life work includes specific external behaviours such as shopping and cleaning, and internal behaviours such as managing stress, anxiety and psychological emotions (Wang et al., 2015).

Previous studies have applied this theory to the long‐term care of long‐term diseases such as metastatic breast cancer (Reed & Corner, 2015), stroke (Mead et al., 2013), primary glioma (Wang, 2016), colon cancer (Zhang, 2018a) and leukaemia (Li, Zhang et al., 2018). For example, in the application to primary glioma, through a phenomenology study, researchers explored intervention measures for different stages of the disease and achieved statistically significant findings. Nevertheless, qualitative studies examining the subjective experience of patients with cerebral infarction are lacking in China. Thus, the purpose of this study is to understand the needs and experiences of older adults with cerebral infarction at different stages of the disease, guided by the long‐term disease trajectory theory. This study serves as a foundation to design tailored interventions to meet the needs of older adults with different stages of cerebral infarction in China. Therefore, our research questions were as follows:

**Research Question 1:** Among older adults with cerebral infarction, what are their needs and experiences during the onset, acute and stable disease stages?

**Research Question 2**: What are the characteristic disease trajectory changes older adults with cerebral infarction experience at different disease stages?

## 2 METHODS

### 2.1 Setting and participants

Participants were recruited from the Department of Neurology at a hospital in Xu Zhou, Jiangsu Province, China. Purposive sampling was used to recruit participants, including patients, doctors and nurses in the neurology department of the participating hospital. Patients were recruited based on the following criteria: (1) patients aged 60 and older with a diagnosis of cerebral infarction based on head CT or MRI; (2) patients who experienced the disease stages discussed earlier and were able to verbalize their feelings. Physicians and nurses were recruited based on the following criteria: (1) neurologists and nurses with at least five years' experience working with patients with ischemic stroke. We recruited 14 older adults with cerebral infarction, three doctors and five nurses. Among the patients, 12 had disease recurrence, had experienced all stages of the disease and could provide information on every disease stage. The other two had their first cerebral infarction and were in the onset stage (Table 1). We stopped data collection when saturation was reached, and no new themes were identified. Then, we interviewed the eight doctors and nurses (Table 2).

**TABLE 1:** Demographic information of participants (*N* = 14)

Number	Gender	Age	Recurrence rate	Education	Medical insurance type
P1	Female	72	1	Primary school	Rural medical insurance
P2	Female	72	0	Illiterate	Town medical insurance
P3	Male	80	2	Bachelor's degree	Employee health insurance
P4	Male	70	3	Primary school	Town medical insurance
P5	Male	65	2	Junior high school	Town medical insurance
P6	Female	62	0	Primary school	Town medical insurance
P7	Female	68	1	Illiterate	Rural medical insurance
P8	Female	74	1	Illiterate	Rural medical insurance
P9	Female	62	1	Primary school	Town medical insurance
P10	Male	80	1	Primary school	Rural medical insurance
P11	Male	73	1	Junior high school	Town medical insurance
P12	Male	66	1	College	Employee health insurance
P13	Female	66	3	Illiterate	Rural medical insurance
P14	Male	71	1	College	Employee health insurance

**TABLE 2:** Demographic information of doctors and nurses (*N* = 8)

Number	Gender	Age	Professional title	Working years	Education
D1	Male	56	Chief physician	30	MD
D2	Male	45	Associate chief physician	22	MD
D3	Male	39	Attending physician	22	MD
N1	Female	36	Nurse‐in‐charge	14	Bachelor's degree
N2	Female	45	Associate chief nurse	22	Bachelor's degree
N3	Female	38	Nurse‐in‐charge	16	Bachelor's degree
N4	Female	27	Nurse practitioner	5	Bachelor's degree
N5	Female	36	Nurse‐in‐charge	13	Bachelor's degree

### 2.2 Data collection

Based on the long‐term illness trajectory framework and Burton's (2000) classification of the trajectory of cerebral infarction, we recruited patients experiencing the onset, acute and stable stages of the disease for this study. The onset stage refers to when the patients exhibit initial signs and symptoms and are diagnosed with cerebral infarction by CT or MRI. The onset stage lasts approximately one to two days. In this study, the acute phase refers to the stage when patients develop severe symptoms and cannot improve on their own, thus requiring hospitalization for about 7–15 days. The stable stage lasts about 1‐6 months after the disease and symptoms are under control and the disease no longer progresses.

Semi‐structured interviews (Polit & Beck, 2017) were conducted from January to May 2019. We developed an interview outline to interview participants with different roles (Table 3). The interview contained open‐ended questions (e.g. “How do you feel?” “What problems did you encounter during the hospitalization?” and “What is the impact of this disease on your psychology and daily life?”), which were used to support the interview process and encourage the participants to elaborate on their responses (Polit & Beck, 2017). The researcher selected the time (i.e. during working hours) and place (i.e. their workplace) for interviews and explained the reasons for recording to the participants. The researcher asked for permission to start the interview after the recording began. To keep the environment quiet and protect the patients' privacy, all interviews took place at a doctor's office and lasted for an average of 35 minutes. Each participant was interviewed once. After each interview, the researcher offered a small gift to the participant.

**TABLE 3:** Interview outline

	Questions
Generic questions for all patients	Where are you uncomfortable now?
	Have you had any previous experience of cerebral infarction? How does this affect your life?
	What problems did you encounter during the hospitalization?
	After leaving the hospital, what are your experiences? What are the needs?
	What is the impact of this disease on your psychology and daily life?
	Can you tell me your experience of going out after cerebral infarction? What activities did you participate in?
	What other support or help do you want?
Generic questions for doctors and nurses	During hospitalization, can you talk about what the elderly patients with cerebral infarction generally have and how do you deal with it?
	What kind of problems will the revisited patients encounter after being discharged? What are your suggestions?
	What personal experience and methods do you think can deal with their problems?
	What advice do you have for inpatient care and long‐term care?

### 2.3 Data analysis

After the interview, the recording was transcribed in 24 hours by two researchers. Colaizzi's descriptive phenomenological method (Morrow et al., 2015) was used to analyse the data. The research team used the Nvivo 12 software to analyse the interview data. They extracted themes by induction, coding and classification and finally returned the results to interviewees for verification. Using the long‐term illness trajectory framework as guidance, we extracted multiple themes and subthemes about the experiences and needs of older adults with different stages of cerebral infarction based on the three dimensions of the long‐term disease trajectory illness‐related work, biographical work and everyday life work. During data analysis, two researchers analysed the data with a memorandum. Conflicts in data coding were resolved through discussions between two researchers or verification from interviewees. A senior researcher was also available to guide the analysis. The analysis notes were taken to ensure the credibility of the results.

### 2.4 Ethics

The Ethical Review Committee of the affiliated hospital of a university approved this study. Written informed consent was obtained from all the participants.

## 3 RESULTS

According to the study design and data analysis, the experience and needs of older adults with cerebral infarction based on the three dimensions of long‐term disease trajectory illness‐related work, biographical work and everyday life work at different stages of the disease were presented below. Table 4 presented the main themes and illness trajectory changes at different stages.

**TABLE 4:** Main themes and illness trajectory changes of different stages

Dimensions	Stages			Trajectory changes
	Trajectory onset period	Acute period	Stable period	
Illness‐related work	Sudden disease symptoms	Unrestored physical functioning	Restored physical functioning	The transition from sudden onset of helplessness to active rehabilitation exercise
	Searching for causes of the disease	Lack of knowledge about the disease management	Adoption of rehabilitation exercises to prevent recurrence	
Biographical work	Lack of biography	Adaptation to disease outcomes	Stabilizing biography	The transformation from lack of biography to reshaping biography
	Adaptation to the new state of life	Acceptance of fate	Reshaping biography	
Everyday life work	Uncertain treatment outcomes	Desire for psychological support	Gradual improvement in daily living	The transition from eager for help to reshaping a healthy lifestyle
	Desire for help	Desire for long‐term care	Reshaping healthy eating habits	

### 3.1 Illness‐related work

#### 3.1.1 Trajectory onset stage

There were three themes related to illness‐related work in this stage: sudden disease symptoms affecting normal functioning, delays in seeking medical treatment and searching for causes of the disease.

##### Theme 1: Sudden disease symptoms affecting normal functioning

After the onset of the disease, most patients developed physical dysfunction that affected their daily life.

Now, my mouth is watering and I can't speak clearly. Sometimes my feet are numb and I can't walk easily. (Patient 10)

The patient may suddenly develop symptoms such as slurred speech, numbness, weakness, unsteady walking, or choking on food. (Doctor 1)

##### Theme 2: Delays in seeking medical treatment

Most patients failed to identify symptoms promptly; thus, the optimal treatment time was delayed, which had a negative impact on their treatment outcomes and quality of life.

When the disease first developed, only my husband and I were home. He noticed that I didn't speak for several days, but he didn't know it was a cerebral infarction. We would have gone to the hospital earlier if we had found it out. By the time we met the doctor, I was already walking unsteadily. (Patient 6)

The patients should go to hospitals and receive treatment in time after being diagnosed with cerebral infarction by doctors. The earlier the patient goes to the hospital, the better the treatment effects. Instead, he waited for the symptoms to become more serious before going to the hospital for treatment, at which point the patient had already missed the best timing for treatment. (Doctor 2)

##### Theme 3: Searching for causes of the disease

The doctors and nurses mentioned that patients with cerebral infarction struggled to identify the aetiology to manage their symptoms timely and effectively.

The patient was admitted to the hospital for treatment of the cause of the disease, with a clear diagnosis and timely control of the development of the disease. (Doctor 1)

The primary treatment for these patients at the hospital is to screen for the cause and find the root cause; for example, whether it is the cardiogenic cerebral infarction or cerebral infarction caused by cerebral arteriosclerosis and thrombosis. If patients have mild strokes, they may have vascular malformations, moyamoya disease, or arterial dissection. Therefore, it is key that clinicians helps patients identify the causes of cerebral infarction and provide them with appropriate measures. (Nurse 1)

#### 3.1.2 Acute stage

Two themes were identified in this stage: unrestored physical functioning and lack of knowledge about disease management.

##### Theme 1: Unrestored physical functioning

Some functions were not restored right away. Many patients were living with speech impairment, cognitive impairment and somatic dysfunction.

At the beginning, I felt dizzy and numb. Then, I vomited and felt panicked and had very few meals. After the treatment, there was no vomiting, only dizziness, numbness in my hands and feet, and no strength in my body. (Patient 1)

I can't speak clearly, and my memories decline. My hands and feet are numb, and I can't walk very well. This has been a long time. (Patient 3)

##### Theme 2: Lack of knowledge about the disease management

Most patients lack knowledge on the prevention of cerebral infarction, healthy diets, rehabilitation measures and nursing care.

I usually know some sequelae of cerebral infarction, such as hemiplegia and numbness of hands and feet, but I don't know how to perform rehabilitation exercises, nor do my family, and no one teaches us how to do rehabilitation exercises. (Patient 1)

I don't pay attention to the disease at ordinary times, but I don't strictly control the diet, and I don't know the dietary requirements of cerebral infarction. We have a local community hospital, but I don't go there for regular physical examinations or anti‐thrombotic treatment. I don't usually strictly follow a doctor's advice on the use of this drug. (Patient 11)

#### 3.1.3 Stable stage

There were two themes in this stage: restored physical functioning and adoption of rehabilitation exercises to prevent recurrence.

##### Theme 1: Restored physical functioning

After continuous treatment and care, the patients regained some limb function but did not achieve the same level of function as before.

At the beginning of my illness, I did not move quickly. My mouth cannot speak. Today, I can walk slowly. At present, my activities are limited. (Patient 7)

After the recurrence of the disease, the handshaking was serious at the beginning, but now it is better, the housework can be done normally. (Patient 5)

##### Theme 2: Adoption of rehabilitation exercises to prevent recurrence

Many patients recovered through rehabilitation training and actively prevented disease recurrence.

My symptoms of hand‐shaking have become worse, affecting my normal eating and speaking. I get dizzy and my legs get heavy in the middle of the day, and then the more I exercise, the less I feel. (Patient 12)

Sometimes I feel dizzy, my legs and feet are unsteady when I walk, and my mouth is a little crooked and drooling. My doctor often asks me to do some exercise. I did physical exercise according to his requirements, which was effective, but my recovery was slow. (Patient 14)

### 3.2 Biographical work

#### 3.2.1 Trajectory onset stage

There were two themes related to biographical work in this stage: lack of biography and concern about disease outcomes.

##### Theme 1: Lack of biography

In the face of sudden disease symptoms, patients were prone to a lack of biography.

I have been unable to move my hands and feet for more than ten days now. Now I am worried about what to do in life if I cannot walk in the future. (Patient 8)

Many patients never expect to get this problem, they used to think that they were quite healthy but were perhaps mistaken. They often ask if they can recover, how long it will take? (Nurse 5)

##### Theme 2: Concern about disease outcomes

Symptoms and signs of the disease made patients feel insecure and unable to adjust to the fact that they were ill.

I was very scared and a little panicked after being diagnosed with cerebral infarction as it was all new to me. (Patient 2)

The patient is most worried about when he can recover, when he can get well, when he can get out of bed, and whether there will be a sequela, such as limping relapse. (Doctor 1)

#### 3.2.2 Acute stage

There were two themes in this stage: adaptation to a new state of life and acceptance of fate.

##### Theme 1: Adaptation to the new state of life

By understanding the disease, the patient accepted the outcome.

The disease has relapsed this time. I'm in a good mood and not under too much pressure. I'm old and can think of lots of things, so I don't have anything to worry about. (Patient 3)

When I was diagnosed with cerebral infarction, I was not afraid, and I was not afraid after the recurrence of the disease. Generally speaking, it had little influence on my life. I always do what I should do and eat what I should eat. (Patient 5)

##### Theme 2: Acceptance of fate

Some patients were unsure about the prognosis of the disease. They showed resignation and let nature take its course.

I think when I get older, I will have some kind of disease or discomfort. There is no use worrying about it, nor thinking about it. Sometimes I think it is better to die than to live. (Patient 1)

#### 3.2.3 Stable stage

There were two themes in this stage: stabilizing biography and reshaping biography.

##### Theme 1: Stabilizing biography

Most patients developed their cognition and attitude toward the disease after recovery and formed a stable biography.

I feel that my physical recovery is OK at present, and I have the confidence to make a better recovery. I maintain a positive attitude every day and maintain a regular life. (Patient 5)

Patients with long illnesses and recurrence discuss and exchange disease experiences with us most of the time and know quite a lot. (Nurse 2)

##### Theme 2: Reshaping biography

After experiencing remission from the disease, most patients developed their cognition and attitude toward the disease and formed a new self‐concept to face reality again.

Since I got sick, I have come to the understanding that I hope to be in good health and capable of doing what I want to do, as long as my physical condition permits. (Patient 14)

After being discharged from hospital, many patients are still active in life, and many patients like to travel and enjoy life after the illness. (Doctor 1)

### 3.3 Everyday life work

#### 3.3.1 Trajectory onset stage

There were two themes related to everyday life work in this stage: uncertainty about treatment outcomes and desire for help.

##### Theme 1: Uncertain treatment outcomes

The prognosis of the disease is full of uncertainty, and patients often could not imagine the impact of the disease on their later life, leading to constant anxiety and fear.

What I am most worried about this time are immobility and hemiplegia, and what I should do if I can't walk or move. (Patient 9)

The main concerns of patients are whether their physical symptoms will not be relieved, or whether they will always need family care. (Doctor 3)

##### Theme 2: Desire for help

Patients feared that the disease would lead to a negative outcome and were eager to get help and guidance from medical staff.

I don't know much about cerebral infarction, and I don't know how to treat it. Can I be cured? (Patient 2)

Patients still have many questions about our nursing work, and they often consult us about the treatment and nursing process related to the disease. (Nurse 4)

#### 3.3.2 Acute stage

There were three themes in this stage: desire for psychological support, desire for long‐term care and desire to improve sleep quality.

##### Theme 1: Desire for psychological support

Most patients experience feelings of fear, depression and anxiety about the complications, recurrence and prognosis of cerebral infarction and have a strong sense of uncertainty.

I have a cerebral infarction this time, and now I feel that it is better to die than to live. From the first attack to this relapse, I was under great pressure and had poor sleep quality. Now I am under too much mental pressure. I often feel fear, tension, and anxiety, which affects my normal life and my family. If I could exercise well, I would be very active. (Patient 8)

Patients with recurrence may have new problems, such as anxiety and depression after repeated attacks of the disease. We may need to focus on these issues. (Nurse 3)

##### Theme 2: Desire for long‐term care

Many patients lose the ability to take care of themselves and need long‐term assistance.

I cannot perform a series of housework such as sweeping the floor, and my ability to take care of myself is poor. I need others' long‐term help to walk. (Patient 3)

The patient was not at home alone and forgot to take medicine for several days. He has not taken any medicine at all, and his memory has declined. He needed long‐term help. (Nurse 2)

##### Theme 3: Desire to improve sleep quality

Many older people had poor sleep quality and sleep quality dropped further after cerebral infarction.

Now I sleep badly. First, I can't fall asleep or sleep for no more than one or two hours. Therefore, I have little or no energy. I'm desperate to improve my sleep. (Patient 3)

My sleep quality is so bad that I can't keep my spirits up every day for two hours a day. How can I really sleep? (Patient 4)

#### 3.3.3 Stable stage

There were three themes in this stage: gradual improvement in daily living, decreased social connections and reshaping healthy eating habits.

##### Theme 1: Gradual improvement in daily living

The patients' normal functioning was gradually restored, and daily life slowly returned to the state before the illness with continuous medical care and rehabilitation.

I try to do housework, and now I can do a little bit. I like to walk when I exercise, and I think walking is good for my whole body. (Patient 12)

I like swimming and playing basketball. Now I often go to Yunlong Lake to swim at about 5 pm. The lake water is quite warm in the daytime, so swimming is enjoyable. (Patient 13)

##### Theme 2: Decrease social connection

The weakened social communication ability of older adults escalated the process of cognitive impairment and depression after stroke.

After my cerebral infarction, my social communication decreased, and I did not make as many friends as before, which largely affected my social life. We used to have many friends, but now we are old and don't see each other much. (Patient 9)

My personality has changed a lot, and I speak less than before. I used to like visiting relatives and friends and traveling around the country on business. I enjoyed traveling and making friends. I have a lot of friends in the literary and sports circles, and even around the country, I have friends, but now we basically do not have contact. (Patient 14)

##### Theme 3: Reshaping healthy eating habits

In the interview, many patients did not know much about the cerebral infarction diet, and almost all of them had suboptimal lifestyles. After the disease, they strove to change their eating habits and were eager to restore a healthy diet.

I have given up smoking and drinking since my first illness, and I am on a light diet now, so I dare not eat big meals with extensive salt and oil. (Patient 5)

I like smoking and drinking, and I'm trying to quit them, but I don't think smoking and drinking have much impact on me. I usually eat salty and spicy food; I don't think that the cerebral infarction significantly affects my daily diet. (Patient 10)

## 4 DISCUSSION

The treatment, nursing and rehabilitation of cerebral infarction are complex. We found that the experiences and needs of older adults at different stages of cerebral infarction changed dynamically, which is consistent with prior findings (Davoody et al., 2016).

This study indicated that patients have different experiences and needs of illness‐related work. Symptoms were more severe in the trajectory onset stage than in other stages. Therefore, when patients were in this stage, clinicians should actively look for the cause of cerebral infarction and help them manage symptoms. Moreover, given the opportunity of remission of disease symptoms, clinicians should encourage patients to cooperate with them about treatment and disease management in the acute stage and help them recover (Farahani et al., 2020). As for the stable stage, rehabilitation training was necessary to prevent recurrence for patients left with sequelae of cerebral infarction. Healthcare providers should pay attention to the patients' needs, provide personalized programs and support, relieve the symptoms of discomfort and help the patient recover physical functioning.

Our study also showed that patients' demand for knowledge of illness‐related work increased, and the content changed dynamically. In the trajectory onset stage, patients mainly desired knowledge about the meaning of the disease diagnosis and disease outcomes. While in the acute stage, patients had more knowledge and desired for medical treatment, nursing care, healthy diets and rehabilitation. During the stable stage, patients were more interested in knowledge about the prevention of disease recurrence and developing a healthy lifestyle. Previous studies in China showed that a lack of understanding of the disease made patients fearful, uneasy and uncertain (Benoit et al., 2020). Disease management knowledge helped patients alleviate fear (Fukuoka et al., 2019), prevent disease recurrence and promote physical health (Ram et al., 2017). Therefore, clinicians should closely communicate with patients and their families and provide education about the disease, treatment, rehabilitation and recommended lifestyles. Patients need to understand the harms of recurrent cerebral infarction, increase their self‐protection awareness and avoid the risk factors associated with recurrence.

Patients also have distinctive and dynamically changed demands for biographical work. Our study showed that patients' biographical work was absent in the onset and acute stages; some patients would go with the flow and accept their fate, gradually stabilizing their self‐concept in the stable stage. A positive self‐concept could reduce the risk of depression and promote patients' confidence in facing the disease (Wang, Liu et al., 2020). Therefore, we recommend that clinicians pay attention to the dynamic changes in patients' biographical work, assisting them in establishing positive biographical work, helping them adapt to the disease outcomes, relieving their negative emotions and guiding them to face the disease positively. Currently, nursing care models do not include patients' biographical work in China. At the same time, the application of the long‐term illness trajectory theory has a positive effect on patients' illness‐related work, biographical work and everyday life work (Wang, 2016). Therefore, this theory can be applied to the care of older adults with cerebral infarction to promote patients' adaptation to the disease outcomes and encourage them to form positive biographical work.

Patients have distinctive demands of everyday life work, which tend to change dynamically. Experience in daily life is conducive to overcoming mental illness, maintaining normal activity abilities and stabilizing self‐concept (Nunes & Queirós, 2017). During the trajectory onset and the acute stage, the daily living ability of patients is poor due to the symptoms of the disease, leading to heavy psychological pressure and family‐related burdens. During the stable stage, the daily living ability of the patients gradually recovered, and some patients had sequelae and needed rehabilitation training. At the same time, our study showed that cerebral infarction in older adults could influence their mental state: positive aspects such as hope and confidence, and negative aspects such as fear, tension, anxiety and depression. However, most patients exhibited psychological symptoms. This result was consistent with a study by Li, Xiong et al. (2018). They proposed that after strokes, patients with cerebral infarction can experience depression, anxiety and other psychological symptoms that negatively impact their daily lives. This study also showed that patients' dietary habits affected disease recurrence, which was consistent with a study by Zhang (2018b). A healthy diet is critical in preventing cerebral infarction recurrence. Therefore, clinicians should promote patients' healthy lifestyles, initiate dietary education and work with patients to develop personalized nutrition plans.

### 4.1 Study limitations

The limitation is that this study was only conducted in one hospital in China. Future studies are needed to include broader geographic areas in China, particularly rural areas, to understand the experiences and needs of older patients with cerebral infarction in rural areas in China.

## 5 CONCLUSION

In this study, we explored the experiences and needs of older adults at different stages of cerebral infarction based on the three dimensions of the long‐term disease trajectory illness‐related work, biographical work and everyday life work, which tend to change dynamically over the course of their disease. Our findings lay the foundation for clinicians to develop tailored interventions and treatment plans based on older adults' experiences and needs at different disease stages.

## CONFLICT OF INTEREST

The authors declared no conflict of interest.

## ETHIC STATEMENT

The study was approved by the ethics review committee of the Affiliated Hospital of Xuzhou Medical University (XYFY2018‐KL038). Participants were informed verbally and in writing that their information would remain confidential. They were also informed on their right to withdraw from the study at any time, without needing to provide a reason.

## Data Availability

The data that support the findings of this study are available on request from the corresponding author. The data are not publicly available due to privacy or ethical restrictions.
